# Supplementary material for: Hybrid Models and Biological Model Reduction with PyDSTool
Source: PLoS Comput Biol. 2012 Aug 9;8(8):e1002628. doi: 10.1371/journal.pcbi.1002628 (PMC3415397; doi:10.1371/journal.pcbi.1002628)
Supplement: Text S4 — Complete source code for the PyDSTool package (version 0.88.120504). Includes API documentation and help files linking to web pages. This file is identical to the current public release on Sourceforge.net. (ZIP) [file pcbi.1002628.s004.zip › PyDSTool/html/toc-PyDSTool.PyCont.TestFunc-module.html]

xml version="1.0" encoding="ascii"?


TestFunc


# Module TestFunc

---

## Classes

AddTestFunction  
 BT\_Fold  
 BT\_Hopf  
 BT\_Hopf\_One  
 B\_Check  
 BiAltMethod  
 BorderMethod  
 Branch\_Bor  
 Branch\_Det  
 CP\_Fold  
 DH\_Hopf  
 DiscreteMap  
 FixedPointMap  
 Fold\_Bor  
 Fold\_Det  
 Fold\_Tan  
 Function  
 GH\_Hopf  
 GH\_Hopf\_One  
 Hopf\_Bor  
 Hopf\_Det  
 Hopf\_Double\_Bor\_One  
 Hopf\_Double\_Bor\_Two  
 Hopf\_Eig  
 LPC\_Det  
 NS\_Det  
 PD\_Det  
 ParTestFunc  
 TestFunc  
 UserDefinedTestFunc  

## Variables

\_classes

---

[hide private]
